# Chronic stepwise cerebral hypoperfusion differentially induces synaptic proteome changes in the frontal cortex, occipital cortex, and hippocampus in rats

**DOI:** 10.1038/s41598-020-72868-w

**Published:** 2020-09-29

**Authors:** Vanda Tukacs, Dániel Mittli, Balázs András Györffy, Éva Hunyady-Gulyás, Dávid Hlatky, Vilmos Tóth, Lilla Ravasz, F. Katalin Medzihradszky, Gabriella Nyitrai, András Czurkó, Gábor Juhász, József Kardos, Katalin Adrienna Kékesi

**Affiliations:** 1 ELTE NAP Neuroimmunology Research Group, Department of Biochemistry, Institute of Biology, ELTE Eötvös Loránd University, Budapest, Hungary; 2 Laboratory of Proteomics, Institute of Biology, ELTE Eötvös Loránd University, Budapest, Hungary; 3 Preclinical Imaging Center, Pharmacology and Drug Safety Research, Gedeon Richter Plc., Budapest, Hungary; 4 Laboratory of Proteomics Research, Biological Research Centre, Szeged, Hungary; 5 Department of Biochemistry, Institute of Biology, ELTE Eötvös Loránd University, Budapest, Hungary; 6 Department of Physiology and Neurobiology, Institute of Biology, ELTE Eötvös Loránd University, Budapest, Hungary

**Keywords:** Diseases of the nervous system, Molecular neuroscience, Neuroscience

## Abstract

During chronic cerebral hypoperfusion (CCH), the cerebral blood flow gradually decreases, leading to cognitive impairments and neurodegenerative disorders, such as vascular dementia. The reduced oxygenation, energy supply induced metabolic changes, and insufficient neuroplasticity could be reflected in the synaptic proteome. We performed stepwise bilateral common carotid occlusions on rats and studied the synaptic proteome changes of the hippocampus, occipital and frontal cortices. Samples were prepared and separated by 2-D DIGE and significantly altered protein spots were identified by HPLC–MS/MS. We revealed an outstanding amount of protein changes in the occipital cortex compared to the frontal cortex and the hippocampus with 94, 33, and 17 proteins, respectively. The high alterations in the occipital cortex are probably due to the hypoxia-induced retrograde degeneration of the primary visual cortex, which was demonstrated by electrophysiological experiments. Altered proteins have functions related to cytoskeletal organization and energy metabolism. As CCH could also be an important risk factor for Alzheimer’s disease (AD), we investigated whether our altered proteins overlap with AD protein databases. We revealed a significant amount of altered proteins associated with AD in the two neocortical areas, suggesting a prominent overlap with the AD pathomechanism.

## Introduction

The common clinical symptom of the diverse group of dementias is the severe deterioration of higher-order cognitive functions^1,2^. The two most common types of dementia are Alzheimer’s disease (AD) and vascular dementia (VD)^3^, which could also coexist, and chronic cerebral hypoperfusion (CCH) could be an important risk factor for AD. There are several models of CCH, such as bilateral common carotid artery occlusion (BCCAO)^4,5^, bilateral carotid artery stenosis (BCAS), or four-vessel occlusion (4VO)^6–8^. We decided to use the stepwise BCCAO model because it provides a more robust effect in reducing cerebral blood flow than the BCAS model. At the same time, we wanted to use a model applicable to the study of long-term chronic effects. Previously, we performed the stepwise occlusion of the two carotid arteries for the gradual development of CCH in the rat brain^9,10^. The consequences of CCH in this model are widely studied; these involve oxidative stress, inflammatory reaction, and ultrastructural aberrations of capillaries^4,5^. Moreover, the development of the molecular hallmarks of AD, such as enhanced tau hyperphosphorylation and β-amyloid level was confirmed in rat brain after CCH induction^11,12^.

At the molecular level, memory trace formation, storage, and retrieval are based on synaptic mechanisms^13^. Synapse loss has been observed in the early stages of neurodegenerative diseases, which correlated with the progression of dementia^14,15^. Therefore, investigation of cognitive decline-related molecular changes is particularly important on synaptosome samples. The hippocampus and frontal cortex are responsible for spatial and working memory as well as cognitive functions, which are highly impaired in AD or VD^16–19^. In the present proteomic study, we investigated the synaptic protein changes of hippocampus and frontal cortex with gel-based two-dimensional differential gel electrophoresis (2-D DIGE).

BCCAO also induces chronic ocular ischemia as the blood supply of the highly vascularized rodent retina comes from the internal carotid artery^20^. The retrograde neurodegeneration of the visual system results in the dysfunction of cortical neurons^21,22^. It could cause extensive structural remodeling in the visual cortex affecting the hypoperfusion-induced changes of the cortical proteome. To study the functional impact of the applied hypoperfusion model on the retina and visual cortex, we performed electroretinogram (ERG) and visual evoked potential (VEP) recordings using retrobulbar light stimulation on freely moving Wistar rats^23^. In order to distinguish the impacts of reduced visual input from the direct effects of cortical hypoperfusion, we did not investigate the whole cerebral cortex but separately the occipital cortex in addition to the hippocampus and frontal cortex.

The synaptic proteome is a complex multi-protein network; thus, its global investigation requires high-throughput technologies measuring hundreds or thousands of proteins simultaneously. The proteomic analysis of synaptosome samples was performed using 2-D DIGE proteomics technique combined with high-performance liquid chromatography–tandem mass spectrometry (HPLC–MS/MS) analysis. This technique is more sensitive to the structural proteins^21,24^, while our previous label-free nanoUHPLC-MS/MS method detects receptor proteins abundantly^25^.

Application of the CCH model for AD research is limited by the lack of studies comparing the known molecular events of AD to the CCH model. As we previously described for an anxiety mouse model^26^, we can compare the obtained proteomics results to the databases collecting the current knowledge about molecular mechanisms of the human disease. The proteome changes in the three cerebral areas were compared to each other and bioinformatic databases of AD. Thus, we could estimate the molecular overlap between the diseases and the hypoperfusion-induced changes in synaptic proteostasis. In this study, we aimed to perform an unbiased experiment on the effects of CCH and quantify the molecular overlap with AD-related proteins. At the same time, we hypothesized that the studied brain areas might show different molecular changes and undergo proteomic alterations comparable to AD-associated pathology.

## Results

### Effective occlusion of common carotid arteries as evidenced using magnetic resonance angiography

**Figure 1:**
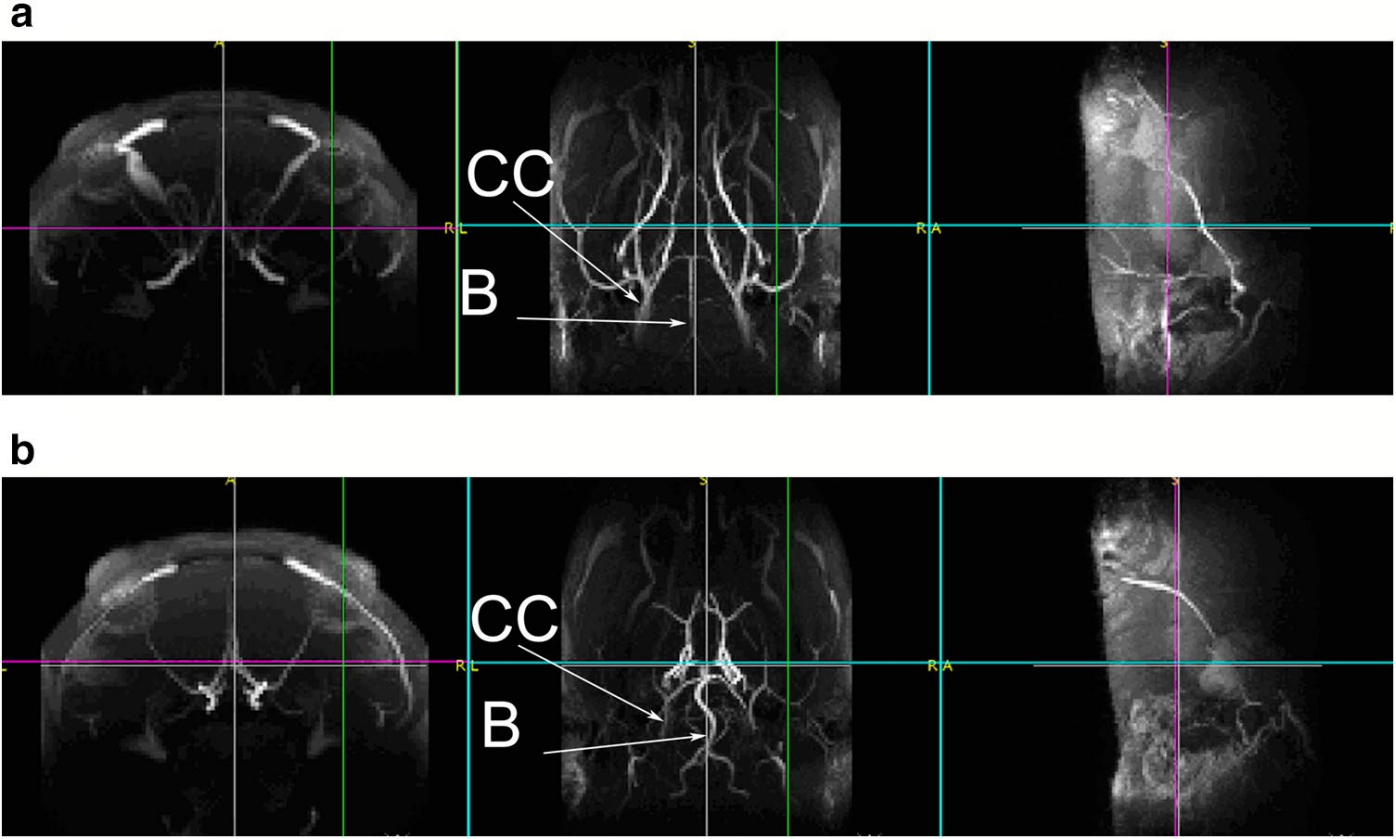
MRI validation of common carotid artery ligation 5 weeks after the occlusions. Representative image from horizontal view of a sham-operated rat (**a**) and an operated rat (**b**) (CC: *arteria carotis communis*; B: *arteria basilaris*). After the occlusion of common carotid arteries, the basilar artery was thickened (**b**).

MRA confirmed that the blood flow was bilaterally blocked in the common carotid artery in each CCH animal, while the sham-operated animals were not affected by the experiment. As a result of the occlusions, the basilar artery is visibly thickened in the treated rats (Fig. 1), suggesting effective BCCAO and increased blood flow in the basilar artery.

### The functional effect of stepwise BCCAO on the visual system

**Figure 2:**
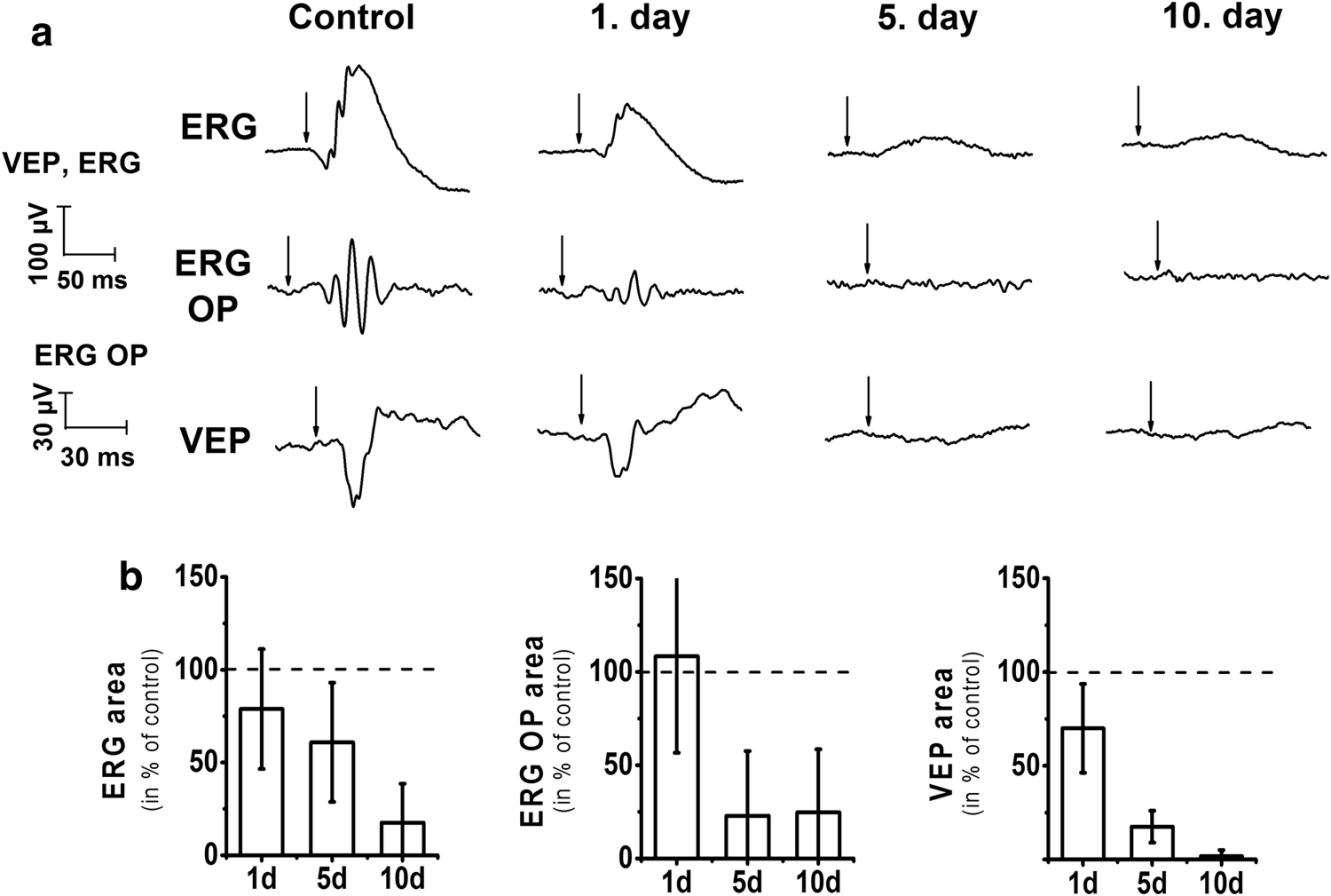
Electroretinogram (ERG), visual evoked potential (VEP), and oscillatory potential (OP) signals before the first, then 1, 5, and 10 days after the second occlusion. Representative recordings of one animal (**a**). The arrows indicate the onset of the 5 ms long light stimulations. The effect of BCCAO on the area of ERG b-wave, OP, and VEP N peak (**b**). The peak areas of recorded signals relative to the control values ± SD are shown. 100% is indicated by a dashed line. The variation between animals in the absolute control values were 23%, 21% and 24% for, ERG b-wave, OP, and VEP N peak, respectively.

Electrophysiological measurements were carried out on 4 rats to study the impact of CCH on the retina and the primary visual cortex. Representative recordings of VEP, ERG, and filtered oscillatory potential (OP) signals are shown in Fig. 2a. Most of the measured parameters declined over time in our experiment (Fig. 2b); however, the SD values were high due to the low number of subjects. The area of ERG b-wave gradually decreased over time (79.1 ± 30.4%, 61.1 ± 32.4%, and, 35.5 ± 15.3% of the control value) 1, 5, and, 10 days after the occlusions. However, the decline of b-wave was not significant. The area of ERG OP signals showed significant (*P* = 0.019) alterations in our experiment. It showed a modest change on day 1 (107.7 ± 61.2%) but declined markedly to 30.5 ± 37.4% and 4.7 ± 9.3% after 5 and 10 days, respectively. Finally, a significant reduction (*P* = 0.007) in the area of VEP N peak signals was apparent. It declined mildly on day 1 (71.3 ± 25.4%), but later on day 5 and day 10 after the operation, it showed a pronounced drop to 17.6 ± 8.2% and 2.7 ± 3.2% of the control level, respectively.

### General summary of protein changes: widespread alterations in the occipital cortex

Proteomic experiments were performed on frontal and occipital cortical and hippocampal synaptosome samples. On the gels, we observed 843, 1072, and 1092 protein spots from the above brain areas, respectively. In the frontal cortex, 27 spots showed significant changes, among them, 11 increased and 16 decreased in the CCH rats in comparison with the sham-operated ones. Strikingly, 67 significant spot changes were observed in the occipital cortex. Out of the 67 altered spots, 43 increased and 24 decreased. In contrast, only 15 spots changed significantly in the hippocampus, as we found 7 spots with increased and 8 with decreased fluorescence intensities (Supplementary Fig. S2). The protein content of spots of interest was identified via LC–MS/MS, which uncovered 33, 94, and 17 altered proteins from the frontal cortex, occipital cortex, and hippocampus, respectively (Supplementary Table S1). The lack of complete correspondence between the number of altered spots and the identified proteins is attributed to the well-known fact that more than one protein could be identified in a spot, one protein can be present in multiple spots, and picked low-abundance spots may lack proteins meeting the identification criteria. Collectively, our high-throughput studies demonstrated the vast extent of proteomic re-adjustment of the occipital cortex due to the CCH. Moreover, we observed modest changes in the frontal cortex and a surprisingly lower proteomic influence of CCH on the hippocampus.

### Impaired cortical energy metabolism and protein turnover in the frontal cortex and cytoskeleton in the occipital areas

**Figure 3:**
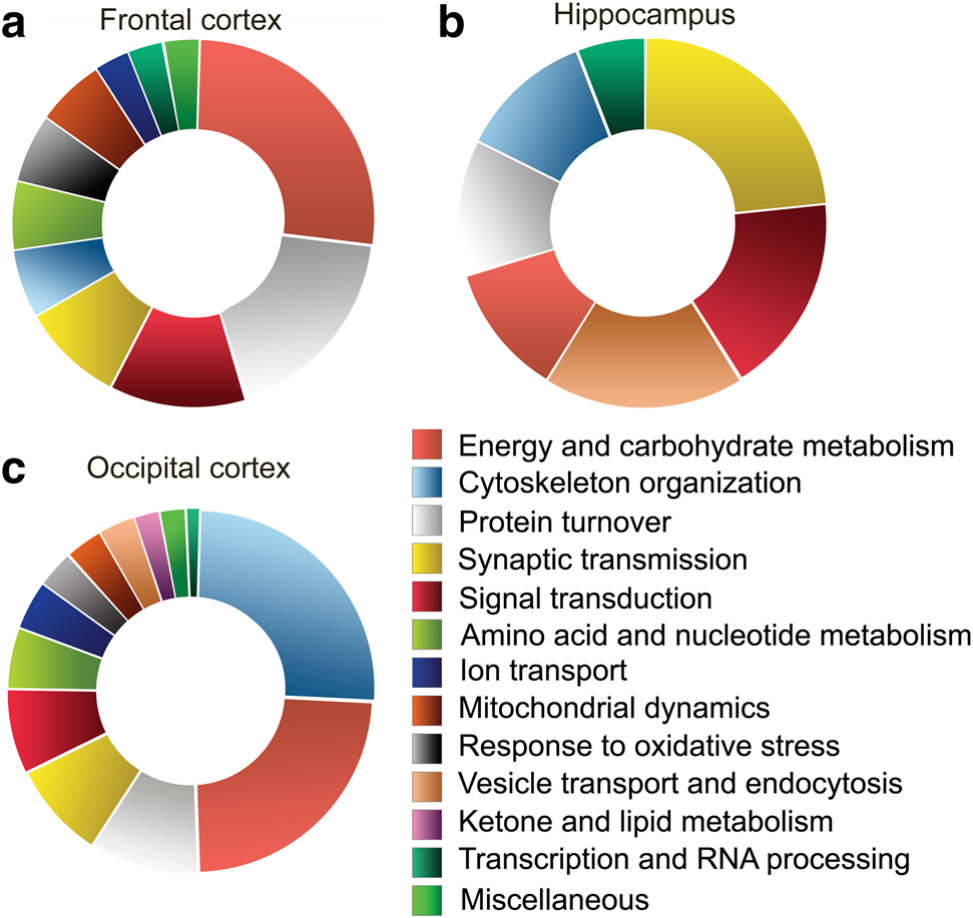
Functional clusters of altered proteins in the frontal cortex (**a**), hippocampus (**b**), and occipital cortex (**c**).

To reveal the most affected synaptic mechanisms by the impaired cerebral blood flow, we carried out functional clustering of the identified proteins (Supplementary Table S2). In the frontal cortex, altered proteins were enriched that are playing role in energy and carbohydrate metabolism (n = 9; 27.3%) and protein turnover (n = 6; 18.2%), followed by functional groups related to signal transduction (n = 4; 12.1%), synaptic transmission (n = 3; 9.1%), cytoskeletal organization (n = 2; 6.1%), oxidative stress response (n = 2; 6.1%), mitochondrial dynamics (n = 2; 6.1%), amino acid and nucleotide metabolism (n = 2; 6.1%), transcription and RNA processing (n = 1; 3.0%), ion transport (n = 1; 3.0%), and miscellaneous (n = 1; 3.0%) (Fig. 3a). In the hippocampus, altered proteins were related to signal transduction (n = 3; 17.6%), synaptic transmission (n = 4; 23.5%), energy and carbohydrate metabolism (n = 2; 11.8%), cytoskeletal organization (n = 2; 11.8%), vesicle transport and endocytosis (n = 3; 17.6%), protein turnover (n = 2; 11.8%), and transcription or RNA processing (n = 1; 5.9%) (Fig. 3b). The most highly represented functional category linked to the altered proteins in the occipital cortex was, interestingly, cytoskeletal organization (n = 24; 25.5%), followed again by energy and carbohydrate metabolism (n = 22, 23.4%). Additional clusters were identified, namely, protein turnover (n = 9; 9.6%), signal transduction (n = 7; 7.4%), synaptic transmission (n = 8; 8.5%), amino acid and nucleotide metabolism (n = 5; 5.3%), ion transport (n = 4; 4.3%), mitochondrial dynamics (n = 3; 3.2%), vesicle transport and endocytosis (n = 3; 3.2%), oxidative stress response (n = 3; 3.2%), ketone and lipid metabolism (n = 2; 2.1%), transcription and RNA processing (n = 2; 2.1%), and miscellaneous (n = 2; 2.1%) (Fig. 3c). Together, our data implied disturbances in synaptic energy homeostasis and protein turnover in the frontal cortex. Additionally, data pointed to synaptic cytoskeletal alterations accompanied by the impairment of energy metabolism in the occipital cortex. On the other hand, the lower extent of proteomic changes in the hippocampus resulted in a homogeneous distribution of proteins among the functional groups with the lack of outstanding enrichment of any of them.

### Protein changes in glucose metabolism and energy production

Considering the enrichment of proteins involved in energy metabolism within our data, we sought to evaluate the results further. We found mostly downregulation of proteins participating in either the citric acid cycle or the electron transport chain. Most importantly, glucose metabolism enzymes were mainly downregulated in the frontal cortex. Fructose-bisphosphate aldolase C (Aldoc), ATP synthase subunit alpha (Atp5f1a), isocitrate dehydrogenase [NADP], cytoplasmic (Idh1), and NADH dehydrogenase [ubiquinone] 1 beta subcomplex subunit 10 (Ndufb10) decreased. In contrast, only glyceraldehyde-3-phosphate dehydrogenase (Gapdh) and pyruvate kinase (Pkm) showed increased levels in this area. Among the rate-limiting enzymes of the citric acid cycle, we detected downregulated isocitrate dehydrogenase [NAD] subunit beta, mitochondrial (Idh3b) abundance in the occipital cortex together with fructose-bisphosphate aldolase A (Aldoa), Aldoc, and citrate synthase (Cs). On the other hand, several other enzymes increased, namely aconitate hydratase, mitochondrial (Aco2), dihydrolipoyl dehydrogenase (Dld), Pkm, pyruvate dehydrogenase E1 component subunit alpha (Pdha1). Finally, an increase in NADH dehydrogenase [ubiquinone] 1 alpha subcomplex subunit 10 (Ndufa10) and a decrease in phosphoglycerate mutase 1 (Pgam1) levels were observed in the hippocampus.

### Coro1a, Pdia3, and Snca were detected with high-level changes in all the examined brain areas

Evaluation of our proteomic dataset unveiled coronin-1A (Coro1a), protein disulfide-isomerase A3 (Pdia3), and alpha-synuclein (Snca) as proteins changing both in the neocortical structures and hippocampus. In addition, they showed an outstandingly high-level alteration in several spots compared to most detected fold changes (Supplementary Table S1). While Coro1a was identified with a ~ twofold increased abundance in all brain areas investigated, Pdia3 was detected in various spots showing moderate, in addition to extremely high and low changes. Finally, Snca was detected with an almost twofold elevation in the occipital cortex and a downregulated state in the frontal cortex and the hippocampus. Altogether, among the wide array of altered proteins, we identified Coro1a, Pdia3, and Snca as proteins most likely to be affected by functional disturbances triggered by CCH.

### Altered proteins overlap with AD databases

In order to estimate the reliability of the CCH model to mimic characteristics of the human diseases, we compared our proteomic data to collected AD protein lists of the DisGeNet and Pathway Studio databases. In the frontal cortex, 36.4% and 45.5% of the altered proteins were associated with AD in DisGeNet and Pathway Studio, respectively. In the occipital cortex, 31.9% and 37.2% of the altered proteins were linked to AD in DisGeNet and Pathway Studio, respectively. In the hippocampus, 41.2% of the altered proteins were found in the AD database of Pathway Studio. The extent of overlap was not significant with DisGeNet (29.4%).

We linked altered proteins taking part in AD with several pathways to unveil affected cellular processes. Not surprisingly, the highest portion of the proteins belongs to the apoptotic degeneration in the occipital and frontal cortices, 91% and 93%, respectively. Enrichment of apoptotic proteins was also high in the hippocampal data, 83% of them were present in the AD-related proteins of the Pathway Studio. In conjunction with the high number of apoptosis-related proteins, cell death-related ones were also highly represented in the group of AD overlapping proteins. However, the representation of memory-related proteins in the AD overlapping protein group showed 47%, 60%, 67% in the occipital cortex, frontal cortex, and hippocampus, respectively. Summarizing the results of overlap, we revealed that our currently described synaptic proteome changes significantly overlap in the frontal and occipital cortices with both AD databases. The overlapping proteins represented the apoptotic and cell death networks highly in all the investigated cortical areas.

## Discussion

In our study, we confirmed the hypoperfusion-induced damage of the visual system in accordance with previous literature^27–29^. In addition, we detected a different extent of proteome alteration in the three brain areas. We found the most widespread changes in the occipital cortex where most of the altered proteins were cytoskeletal. While in the areas responsible for cognitive and mnemonic functions, the frontal cortex and hippocampus, most of the altered proteins were related to energy metabolism and synaptic transmission, respectively. Furthermore, a considerable fraction of identified proteins is also known in the molecular pathomechanisms of AD.

### Benefits and limitations of the study

High-throughput proteomics, in this case, 2-D DIGE is a powerful tool to follow changes simultaneously in the levels of potentially thousands of proteins upon various treatments on animals because it enables effective and reproducible separation of proteins. 2-D DIGE allows the analysis of two or three protein samples simultaneously on a single gel; thus, the same proteins from different samples comigrate as single spots. Accordingly, the superimposition of separate gels with different samples is not necessary, making spot comparison and protein quantification much more reliable than traditional approaches. However, the method has some limitations that must be taken into account. In our study, we separated proteins in the range of approximately 10–100 kDa with isoelectric points (pI) from ~ 3 to ~ 10 pI. Although most of the proteins in a cell fall into these ranges, we could not separate and identify the higher molecular weight proteins and the strongly acidic or alkaline ones. The solubilization of hydrophobic proteins is challenging in general in the field of protein research, such as in 2-D DIGE. It is difficult or even impossible to effectively resolve membrane proteins because they are poorly soluble in the ionic detergent-free conditions applied in isoelectric focusing (IEF).

The first-dimensional separation is based on isoelectric points and the following separation depends on the molecular weight of proteins. Therefore, we are able to detect not only the expression changes of proteins but also the altered levels of protein isoforms, including post-translational modifications (PTM). Consequently, several spots on the same gel may contain the same protein, the alterations of which are most likely caused by PTM. This advantage in resolving power of the method could lead to ambiguities in interpreting and validating protein level changes that could arise from differently regulated protein expression and an altered PTM pattern. Due to these concerns, validation of 2-D DIGE data with traditional orthogonal methods such as western blot and immunohistochemistry is challenging because these tools are generally sensitive only to the expression changes of proteins.

Additionally, the detection of proteins with low abundance is limited. Highly abundant proteins can mask low-abundance proteins' alterations if more than one protein runs into the same spot. This can be improved by subcellular fractionation, such as synaptosome isolation in our current study. However, it is also important to note that contaminations from other cellular organelles are not entirely excluded and present in a non-negligible extent in samples. At the same time, the applied synaptosome preparation is highly reproducible and its power in synaptosome-enrichment was validated several times^25,30,31^.

### Occipital cortex is also affected by the retrograde neurodegeneration of the visual system

We note here that the retina of the rat is highly sensitive to hypoxia^27^, thereby inducing CCH leads to extensive retinal and optic nerve degeneration^28^, which eventually may cause blindness^29^. To provide functional evidence that the BCCAO-induced CCH generates severe malfunctions in the visual system, we performed ERG and VEP recordings on freely moving rats before and after the BCCAO procedure. The marked intensity reduction in VEP on the 5th and 10th day after the BCCAO procedure supports that BCCAO decreases blood circulation in the cortex. However, the non-significant tendency of decline in b-wave suggests the contribution of suppressed retinal input to the VEP reduction. Since the OPs are more sensitive to retinal hypoperfusion than the b-wave^32,33^, the decrease of OPs was significant after hypoperfusion. Thus, the harmful effects of the CCH on the occipital cortex are derived from two sources: (1) from the loss of visual inputs (as confirmed by ERG recordings) and (2) from the reduced cortical blood flow (as suggested by the MRA experiments). The retrograde neuronal degeneration developing in CCH rats likely explains the large differences between the numbers of protein changes in the occipital cortex comprising the primary visual cortex in comparison with the two other structures examined. Based on these observations, we suggest that proteomics studies aiming the protein mechanisms of hypoxia on memory should be performed on other cortical areas than the visual cortex because of the retrograde degeneration of cortical cells by the missing or decreased retinal input^28^. Furthermore, the brain area-specific molecular alterations are masked when we use whole cortex samples for proteomics.

### The CCH model reflects decreased metabolism at the synaptic level

One of the earliest clinical symptoms of AD and VD is the decrease in glucose metabolism in the cortex as measured by fluorodeoxyglucose-positron emission tomography (FDG-PET)^34–37^. Acute hypoperfusion studies revealed a short but intensive change in the cellular metabolism right after the dramatic decrease in oxygen and nutrient availability^4,38^. After a consolidation period, the brain accommodates to the low perfusion rate^5,39^. We emphasize here that as we sacrificed rats eight weeks after the hypoperfusion was started, our data reflect the new homeostatic metabolic state of the synapses and not the acute response to severely decreased blood supply. In the CCH model, glucose metabolism-related protein changes were different in the examined brain areas. We observed a decrease in the amount of glucose metabolism enzymes; among them, isocitrate dehydrogenase (Idh) is one of the rate-limiting enzymes of the citric acid cycle^40^. The decrease in Idh3b in the occipital cortex might be an index of decreased efficiency of the citric acid cycle. A similar decrease in Idh1 was found in the frontal cortex but not in the hippocampus. Possibly, as a compensatory reaction to the impaired citric acid cycle, levels of some of the glycolytic enzymes were increased by hypoperfusion, such as Gapdh, Pkm in the frontal cortex, and Pdha1, Pkm in the occipital cortex. Interesting to note that glucose metabolism enzymes showed a modest change in the hippocampus, while AD causes serious anatomical alterations in the hippocampus and entorhinal cortex^41^. Changes in energy supply in the synapse alter the duration of ATP-dependent repolarization^42^ and the local protein synthesis^43^, leading to an altered proteostasis developed by CCH. The relatively high number of glucose metabolism-related proteins changed in this study indicates that this rat model of dementia can be used for deciphering the metabolic background of AD and VD. These observations raised that the diminished synaptic energy metabolism in rats of the CCH experimental model reflects the decreased cortical metabolism.

### Outstanding protein changes are in correlation with AD

The most extensive changes revealed in all three examined brain regions were Coro1a, Pdia3, and Snca. While there is no direct data on the participation of Coro1a in the pathogenesis of dementias, Pdia3 and Snca are known to be involved in the molecular mechanisms of AD. Pdia3, also called as Erp57, is a disulfide isomerase involved in cytoprotective mechanisms as protein folding regulation^44^. It participates in the amyloid processing pathway in neurons^45^ via, e.g., aiding proper post-translational processing of the amyloid precursor protein^46^. Furthermore, it attenuates ischemia-induced damage by reducing lipid peroxidation and nitric oxide generation^47^. Snca was initially described in the etiology of Parkinson’s disease as an aggregated misfolded protein in synapses^48^. Later, it was uncovered that Snca is present in normal synapses as well^49^, and its increased level was observed in the synapses in AD and AD models^50,51^.

Coro1a is an actin regulator protein widely studied in the immune system as a building block of the immune synapse^52^. Coro1a is present in the neuronal synapse as well and belongs to the group of long-lived proteins^53^. Furthermore, Coro1a was shown to regulate cognitive function via modulating the cAMP/protein kinase A signaling^54^. Mice lacking coronin-1 displayed behavioral abnormalities, including increased aggression, as well as defective learning and memory^55^. Also, in aged senescence-accelerated mice, Coro1a carbonyl levels decreased upon β-amyloid peptide silencing^56^. It is important to note here that all the three proteins’ levels changed in more than one spot on the gels, and the spots were differed by isoelectric point (charge) but not by molecular mass. Phosphorylation is the major and most common contributor to the shifting of protein charge. Interestingly, all Coro1a, Pdia3, and Snca possess several phosphorylation sites (PhosphoSitePlus database^57^) that influence actin-binding and oligomerization properties of Coro1a^58^ and localization, aggregation, and binding affinity of Snca^59^. Therefore, studying the exact change in their phosphorylation state due to CCH-induced pathology possibly represents an important direction of research for a better understanding of the mechanisms of AD and related dementias. In summary, considering that we studied a reestablished, new homeostatic state of the brain under CCH, further investigation of the currently identified highly elevated proteins appears important because they might indicate the reconsolidation of synaptic functions.

### Proteins overlapping with AD represent apoptotic cell death and memory networks

Proteins overlapping with AD are highly represented in apoptotic and memory protein networks. The high representation of apoptosis at the synaptic level was observed in the occipital and frontal cortices as well as in the hippocampus. The apoptotic or necroptotic nature of cell death in AD is not fully established^60,61^, but it is known that proteins of the apoptosis network overlap with the proteins of other cell death types^62^. It suggests that under a chronic hypoperfusion state when the acute phase of hypoxia is over, the brain proteostasis is shifted toward the protein network of apoptosis in different cortical areas. We can conclude that the rat CCH model as a model of neurodegeneration resembles AD partly and highly represents mechanisms of apoptosis. Also, cell death-related proteins are highly represented in the synaptosomal proteome changes of the occipital cortex, where the retrograde degeneration is observed due to the hypoperfusion-induced retinal ischemia.

## Conclusion

In the present study, we conducted gel-based proteomics analyses on synaptosomes prepared from different brain areas of rats subjected to CCH to model dementia via decreased cerebral blood flow. The altered proteins represented essential cellular processes such as energy metabolism, cytoskeletal organization, and protein turnover. We revealed that the occipital cortex shows the most extensive changes in the synaptic proteome among the examined brain areas. However, the widespread molecular adjustment in the occipital cortex is probably not entirely due to the cortical hypoperfusion but also the degeneration of the visual system, as confirmed by our electrophysiological experiments. Nevertheless, the three most extremely altered synaptic proteins (Coro1a, Pdia3, and Snca) were found in not only the frontal cortex and hippocampus but also in the occipital cortex. Each of them is known to be involved in the molecular pathomechanism of AD or impaired cognitive functions. Thus, we do not recommend using cortical tissue samples comprising the visual cortex for dementia research in the CCH model due to the effect of visual degeneration. We provided a bioinformatic estimation of how far this rat model reflects the molecular mechanisms of AD and its animal models. In conclusion, the stepwise BCCAO model can be used to study AD mechanisms besides VD, since several weeks of reduced cerebral blood flow induced overlapping molecular changes with AD mostly in the frontal cortex.

## Methods

### Animals

Adult male Wistar rats (aged 3 months, weighing ~ 220 g) were obtained from Toxi-Coop (Budapest, Hungary). Rats were housed under standard laboratory conditions (lights on at 9:00 AM, lights off at 9:00 PM) in temperature- and humidity-controlled rooms with ad libitum access to food and water. All animal care and experimental procedures were in accordance with the Council Directive 86/609/EEC, the Hungarian Act of Animal Care and Experimentation (1998, XXVIII). They were carried out in strict compliance with the European Directive 2010/63/EU regarding the care and use of laboratory animals for experimental procedures. All the procedures conformed to the National Institutes of Health guidelines were in accordance with the guidelines of the local Animal Care and Use Committee and were approved by the local Ethical Committee of Gedeon Richter Plc. All efforts were carried out to minimize the animals’ pain and suffering and to reduce the number of animals used. The animals were randomly assigned to experimental groups and we assured the experimenters’ blindness to the animal groups whenever possible. A total of 24 rats were used (4 for electrophysiological and 20 for proteomic investigations (n = 14 for CCH and n = 6 for sham operation)) in the experiments.

### Stepwise bilateral occlusion of common carotid arteries

**Figure 4:**
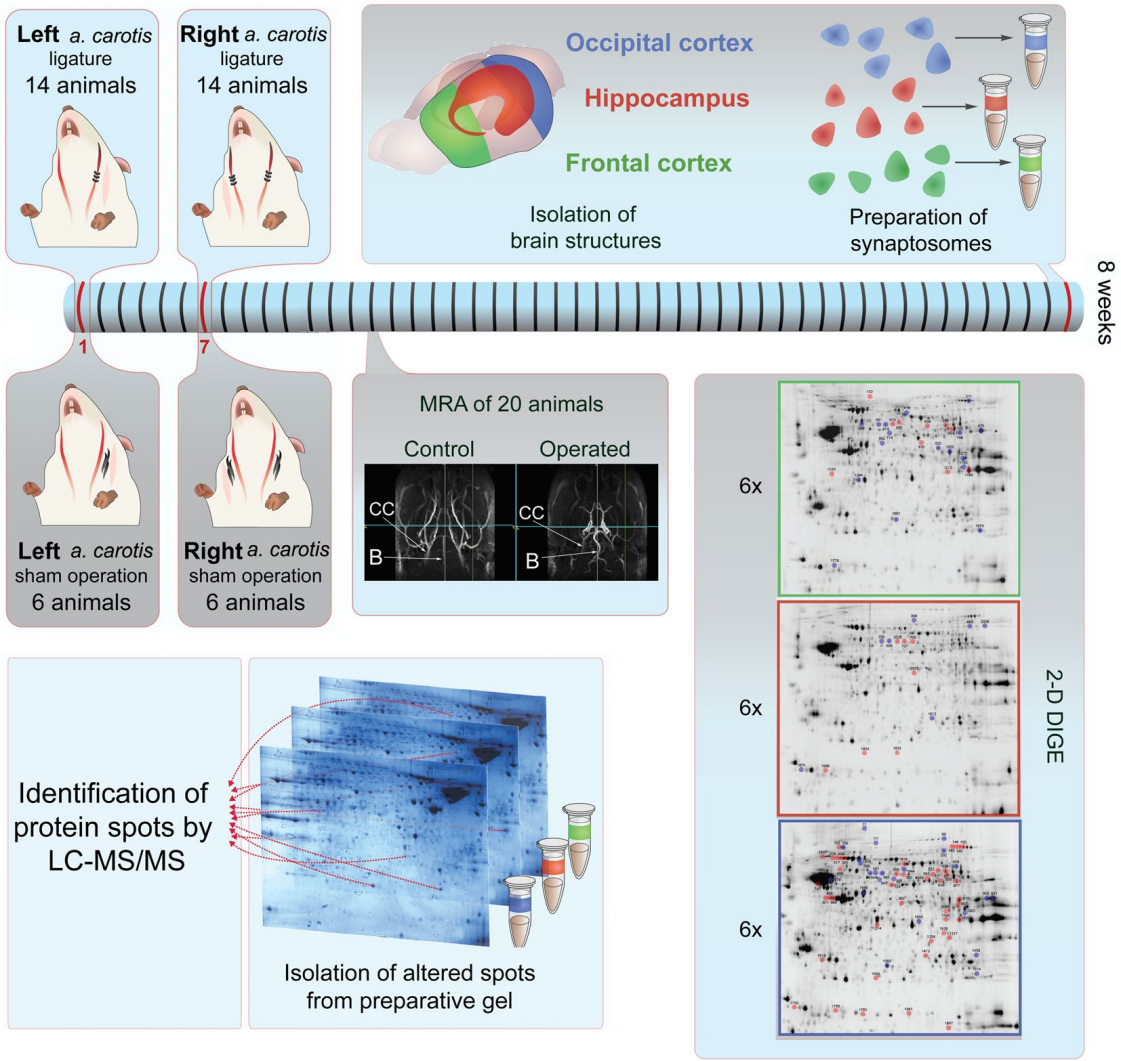
Experimental arrangement of proteomic study. Between the ligature of the left and right carotid artery, animals had one week of regeneration. MRA measurements were performed 2 and 5 weeks after the second occlusion. Animals were sacrificed and three brain structures were isolated from each rat 8 weeks after the second occlusion. Finally, synaptosomes were prepared from the 3 isolated brain regions and 2-D DIGE experiments were conducted.

Stepwise bilateral common carotid artery occlusion was performed on rats as previously described^10,25^. Briefly, rats were anesthetized with isoflurane (1.5–2% in air) and a ventral midline incision was placed on the neck. In the first step, the left common carotid artery was exposed, gently separated from the vagus nerve, and occluded by three ligatures (2-0). After surgery, the animals recovered and were taken to their home cages. In the second step, one week later, the same surgical procedure was performed on the right common carotid artery. Rats in the control group underwent a sham operation as they received the same surgical procedures in both steps, but only a thread was placed around the vessels without ligation of the arteries. Figure 4 shows the experimental arrangement of the proteomic study.

### Electroretinogram and visual evoked potentials

The surgical procedure and electrophysiological recordings were carried out on 4 rats as previously described elsewhere^23^. Briefly, rats were anesthetized with isoflurane (1.5–2% in air) and the skin over the skull was cut in the midline. For recording EEG and VEP, stainless steel screw electrodes were implanted above the right parietal and both primary visual cortices (1 mm lateral from the sagittal suture and 1 mm rostral from the lambdoid suture) and the cerebellar hemispheres (as ground electrodes). For the ERG recordings, we placed a broom-like stainless steel wire over the posterior part of the left eye beneath the upper eyelid. A stainless-steel plate serving as a reference electrode was implanted between the temporal muscle and the skin next to the left ear (Supplementary Fig. S1). All electrodes were soldered to an 8-pin socket that was cemented on the skull together with a stimulus LED connected to a separate 2-pin socket. The red light-emitting LED (maximum emission at 651 nm) was fixed behind the left eye, so the ERG was not influenced by the size of the pupil or by the position of the eye (retrobulbar light stimulation). The rats were allowed a recovery period of 2–3 days before ERG and VEP experiments. Data were recorded on freely moving, dark-adapted rats in a Faraday cage using a BioAmp amplifier (Supertech, Pécs, Hungary) with 0.3 Hz–10 kHz bandpass filtering, a BioStim stimulator (Supertech) and a CED Micro1401-3 data acquisition system (Cambridge, UK) with Signal 4.10 software (Cambridge, UK). We recorded 5 s long frames every 20 s and applied 5 ms long light flashes with 0.1 s prestimulus time (sampling frequency: 15 kHz). The experiment was self-controlled, 50 frames were averaged per trial and we recorded 4–6 trials altogether on 2–3 different days before the first occlusion and 1, 5, and 10 days after the second occlusion. Data were analyzed using the Signal 4.10 and OriginPro 8.5 software by calculating the b-wave area of the ERG and the major negative peak area of the VEP (N peak) recorded from the right primary visual cortex (Supplementary Fig. S1). To examine the fast OP components of the ERG signals, we applied an FFT high pass filter at 40 Hz and calculated the area of the ERG OPs (Supplementary Fig. S1). The changes in VEP, ERG, and OP areas were calculated as the percentage of control results; data are shown as mean ± standard deviation (SD). The statistical analysis was performed by comparing the areas of the signals recorded before and 1, 5, and 10 days after the BCCAO procedure. Friedman ANOVA test was applied (α = 0.05) since the data are not normally distributed and the experimental design is based on repeated measures.

### Magnetic resonance imaging

The efficacy of the occlusions was confirmed with magnetic resonance angiography (MRA) twice for the proteomic experiment based on our previous study^10^. Two and 5 weeks after the second occlusion, anesthetized animals were scanned in a 9.4 T MRI system (Varian, Medical Systems Inc., Palo Alto, CA, USA) with a free bore of 210 mm, containing a 120 mm inner size gradient coil. A 72 mm diameter quadrature-driven birdcage coil (Rapid Biomedical, Rimpar, Germany) was used for RF excitation, and a dedicated 4 channel phased array coil was used for signal reception (Rapid Biomedical). Three-dimensional time-of-flight (TOF) angiography (3D gradient echo) was performed at TR/TE = 40/3 ms, resolution = 0.42 × 0.42 × 0.46 mm. The MRA studies were performed to evaluate cervical and intracranial arteries and parenchymal injury. At 5 weeks an additional T2 weighted anatomical scan was performed (fast spin echo, TR/TE = 15.8 s/28.8 ms, echo train length = 8, matrix = 128 × 128, slices = 150, resolution = 0.17 × 0.17 × 0.2 mm, 8 averages). The MRI recording shown in Fig. 1 was taken 5 weeks after the second occlusion. Experimental animals that were assigned for further experiments had to meet the criteria as follows: (1) lack of gross anatomical abnormalities (i.e., pathologically large or asymmetrical anatomical structures) (2) lack of extensive lesions or any sign of extended tissue impairment. The T2 weighted anatomical scan revealed ischemic lesions in none of the animals; however, one BCCAO rat was euthanized and excluded from the experiment due to the deterioration of its condition.

### Synaptosome preparation

Seven weeks after the second occlusion, rats were deeply anesthetized with isoflurane (1.5–2% in air), and the animals were sacrificed. Their brains were quickly removed from their skulls and rinsed in dry ice-cooled artificial cerebrospinal fluid to remove blood contamination. The cerebral cortices from both hemispheres were separated, then the left hippocampi and the frontal and occipital cortices were dissected on dry ice powder. First, the isolated brain was placed on a pre-cooled Petri dish and cut into two halves in the mid-sagittal plane. A blade was used to cut the anterior part of the brain approximately at the anterior end of the corpus callosum and the frontal cortices were isolated using two forcipes. Subsequently, the hippocampi from both hemispheres were removed using two forcipes. Finally, the occipital cortices at the posterior part of the brain (covering the hippocampus and extending back to the posterior end of the cerebral cortex) were isolated from the inner structures.

The subcellular fractionations were performed immediately after the dissections. The fraction of synaptosomes was prepared following precisely the protocol published by Phillips et al.^63^ and Hahn et al.^64^. Briefly, brain tissues were homogenized in a homogenization solution (320 mM sucrose, 0.1 mM CaCl_2_, 1 mM MgCl_2_) supplemented with protease and phosphatase inhibitor cocktails and homogenized with a Dounce Tissue Grinder (Sigma-Aldrich) manually (40 strokes per sample) at 4 °C with pre-cooled solution and equipment. The sucrose concentration was adjusted to 1.25 M, 1 M sucrose, 0.1 mM CaCl_2_ solution was layered on top of the homogenate, and the samples were ultracentrifuged at 100,000×*g* for 3 h. After ultracentrifugation, synaptosomes were collected from the interface between the two sucrose layers and the proteins were acetone-precipitated overnight at -20 °C. Proteins were resuspended in a lysis buffer (7 M urea, 2 M thiourea, 4% CHAPS, 20 mM Tris, 5 mM magnesium-acetate) and stored at -80 °C.

### Two-dimensional differential gel electrophoresis

The high-throughput investigation of the proteome of the synaptosome samples (6 sham-operated, 6 BCCAO) was performed using two-dimensional differential gel electrophoresis (2-D DIGE). For the separate analysis of the hippocampus, frontal and occipital cortices, three individual runs with 6-6 gels were performed. The samples were adjusted to pH 8.5 and their protein concentration was determined by the 2D-Quant kit (GE Healthcare, Chicago, IL, USA). The fluorescent labeling of the proteins was conducted with a CyDye DIGE Fluor Minimal dye labeling kit (GE Healthcare). Fifty micrograms of proteins of each sample from CCH-treated and control rats were randomly labeled with either Cy3 or Cy5 dyes, while the reference sample (internal standard containing equal protein amounts (25 μg)) was labeled with Cy2. The differently labeled samples were mixed and rehydrated passively onto Immobiline DryStrip gel strips (24 cm, pH 3–10 NL, GE Healthcare) overnight. Isoelectric focusing (IEF) was performed in an Ettan IPGphor 3 IEF unit for 24 h to attain a total of 100 kVh (GE Healthcare). Following IEF, the proteins were reduced and carbamidomethylated using an equilibration buffer containing 1% mercaptoethanol and 2.5% iodoacetamide, respectively. SDS-PAGE separation was performed on 24 × 20 cm, 10% polyacrylamide gels in an Ettan DALTsix Electrophoresis System (GE Healthcare). Then, the gels were scanned with a TyphoonTRIO+ scanner (GE Healthcare) using appropriate lasers and filters with the photomultiplier tube (PMT) biased at 600 V. Differential protein analysis was performed using the DeCyder software package (GE Healthcare), employing its Differential Analysis (DIA) and Biological Variance Analysis (BVA) modules. The fluorescence intensities of the Cy3 and Cy5 dyes on a particular gel were normalized to the intensity of the Cy2 dye. Quantitation of the fluorescence intensities of the protein spots and statistical analyses were carried out using the software. Statistically significantly altered protein spots (*P* < 0.05 using independent, two-tailed Student’s *t*-test) with more than ± 1.15 fold changes (FC) were picked for further protein identification. For the identification of proteins in spots of interest, preparative 2-D gel electrophoresis was performed separately using a total of 800 μg of synaptic proteins per gel. Resolved protein spots were visualized by the Colloidal Coomassie Blue G-250 stain (Merck Millipore, Billerica, MA, USA). One preparative gel for each brain region was run, and the selected spots were manually excised from the gels with pipette tips for protein identification. Excised spots were placed in 1% acetic acid solution.

### Mass spectrometry-based protein identification

Proteins in the selected 2-D gel spots were in-gel digested as described in the protocol available on-line (https://msf.ucsf.edu/protocols.html). Briefly, gel spots were cut to smaller cubes, washed with 25 mM ammonium-bicarbonate/50% acetonitrile, reduced using 10 mM dithiothreitol and alkylated with 55 mM iodoacetamide. After dehydration, the gel pieces were rehydrated with 100 ng Trypsin (sequencing grade, side-chain-protected porcine trypsin, Promega, Madison, WI, USA) in 20 µl of 25 mM ammonium-bicarbonate. Samples were digested for 4 h at 37 °C. Tryptic peptides were extracted and dried in a vacuum centrifuge.

Samples were reconstructed in 20 µl of 0.1% formic acid before mass spectrometric analysis. Five µl of digest was injected for LC–MS/MS analyses onto an LTQ-Orbitrap Elite (Thermo Fisher Scientific, Waltham, MA, USA) mass spectrometer on-line coupled with a nanoAcquity UPLC system (Waters, Milford, MA, USA). In order to shorten the injection time, the sample was injected with high flow rate to a trap column (Symmetry C18, nanoACQUITY UPLC 2D, V/M 0.180 mm × 20 mm, 5 µm, 100 Å, Waters) and a nano column (BEH130, C18 Acquity UPLC M-class peptide column, 0.075 mm × 250 mm, 1.7 µm,130 Å, Waters) was used for the analysis. Gradient elution was applied from 3 to 40% of eluent B (0.1% formic acid in acetonitrile) in 37 min. Mass spectrometry data were collected in a data-dependent manner, and a high-resolution survey scan was followed by a maximum of 5 dependent CID spectra analyzed in the ion-trap. Only multiple charged precursor ions were selected for fragmentation and after, they were excluded for 30 s from the repeated selection. PAVA script (UCSF, MSF, San Francisco, CA, USA) was used for peak picking and our *in-cloud* ProteinProspector (version: 5.22.0) server (https://cloud.mta.hu/) was used for database search, with the following parameters: UniProtKB.2017.9.19.random.concat database was filtered for the human, mouse, rat, and pig sequences (315,955/89,951,742 entries searched). Only tryptic peptides were considered, one missed cleavage and cleavage in front of Pro were permitted. Carbamidomethyl cysteine as fixed and several variable modifications were set as Acetyl (Protein N-term), Acetyl + Oxidation (Protein N-term M), Gln- > pyro-Glu (N-term Q), Met-loss (Protein N-term M), Met-loss + Acetyl (Protein N-term M) and Oxidation (M). Mass accuracy was set to 10 ppm for parent and 0.6 Da for fragment ions. Proteins and peptides were accepted with maximum 1% FDR. Identified proteins were accepted if a minimum of 4 unique peptides and 10% of sequence coverage were accurately identified.

### Functional classification and bioinformatics analysis

Significantly altered proteins were clustered based on the UniProt (https://www.uniprot.org) and GeneOntology (https://geneontology.org) databases as well as with the published literature. The most relevant functions of the proteins were taken into account for clustering. Databases of DisGeNET^65^ and Pathway Studio version 12.1 (Elsevier Life Science Solutions) were employed to discover the extent of overlap between significantly altered proteins in the CCH model and proteins that are known to have a role in the pathomechanism of AD. Modified Fisher’s exact test was utilized to determine the statistical significance of overlaps (EASE score < 0.05). Among the overlapping proteins, involvement in molecular pathways was screened to reveal, which cellular processes were affected. Interactions between overlapping proteins and the involvement of pathways of apoptosis, cell death, and memory were also examined with Pathway Studio 12.1.

## Supplementary information

Supplementary file

## Data Availability

The datasets generated and analyzed during the current proteomics study are available in this published article (and its Supplementary Information file). In addition, the raw MS data is available in the Massive repository, ftp://MSV000085407@massive.ucsd.edu (MSV000085407; password: vanda).
