# Supplementary material for: Chronic stepwise cerebral hypoperfusion differentially induces synaptic proteome changes in the frontal cortex, occipital cortex, and hippocampus in rats
Source: Sci Rep. 2020 Sep 29;10:15999. doi: 10.1038/s41598-020-72868-w (PMC7524772; doi:10.1038/s41598-020-72868-w)
Supplement: Supplementary file 1 — Supplementary file [file 41598_2020_72868_MOESM1_ESM.pdf]

Supplementary information

Chronic stepwise cerebral hypoperfusion differentially induces synaptic proteome changes in the frontal cortex, occipital cortex and hippocampus in rats

Vanda Tukacs, Dániel Mittli, Balázs András Györffy, Éva Hunyady-Gulyás, Dávid Hlatky, Vilmos Tóth, Lilla Ravasz, Katalin Medzihradszky F., Gabriella Nyitrai,András Czurkó, Gábor Juhász, József Kardos, Katalin Adrienna Kékesi

Supplementary Fig. S1 The layout of the head implant (a) and the evaluated parameters of electrophysiological recordings (b). The stimulating LED was cemented behind the left eye. The cortical electrodes were screwed above the primary visual cortices for VEP recording (V1L, V1R), the cerebellar hemispheres as ground electrodes (CL and CR) and the right parietal cortex for background EEG recording (P). The corneal electrode (Cor) was implanted beneath the left eyelid to make proper contact with the surface of the eyeball for ERG recording. The reference electrode (Ref) was placed between the skin and the temporal muscle on the left side facing the skin with its active surface. From the ERG signals, we calculated the absolute area (1) of the b-wave. After applying a 40 Hz high pass filter we summed up the absolute areas (2, 3, 4) of the ERG OPs. From the VEP recording, we calculated the absolute area (5) of the major negative component (N peak)

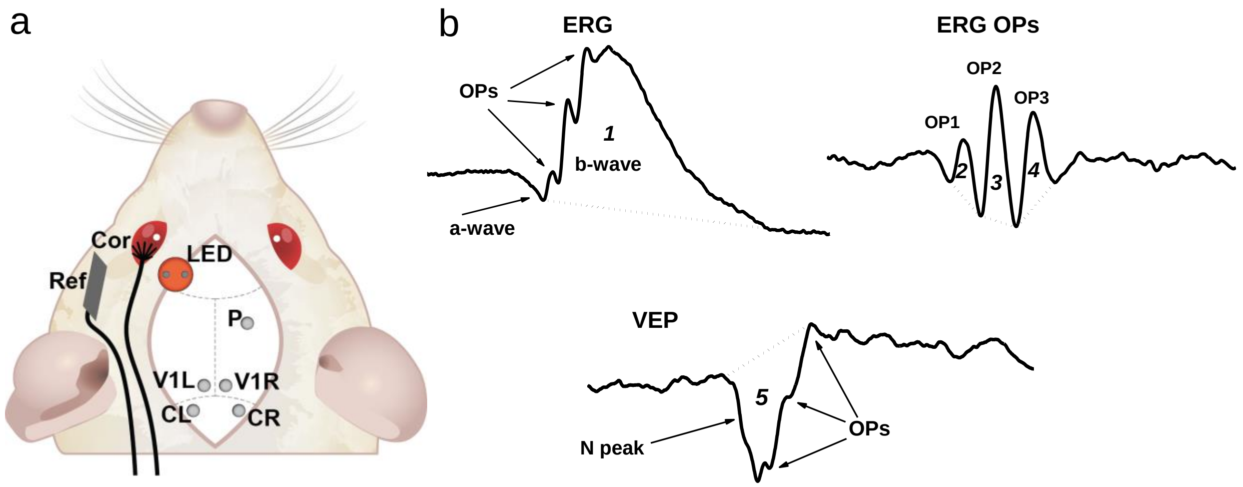

Supplementary Fig. S2 Representative gel images of 2-D DIGE experiments (left) and their corresponding volcano plots (right) of the frontal cortex (a), hippocampus (b), and occipital cortex (c). Significantly altered protein spots (P < 0.05) are marked either with blue (FC ≤ -1.15) or red (FC ≥ 1.15)

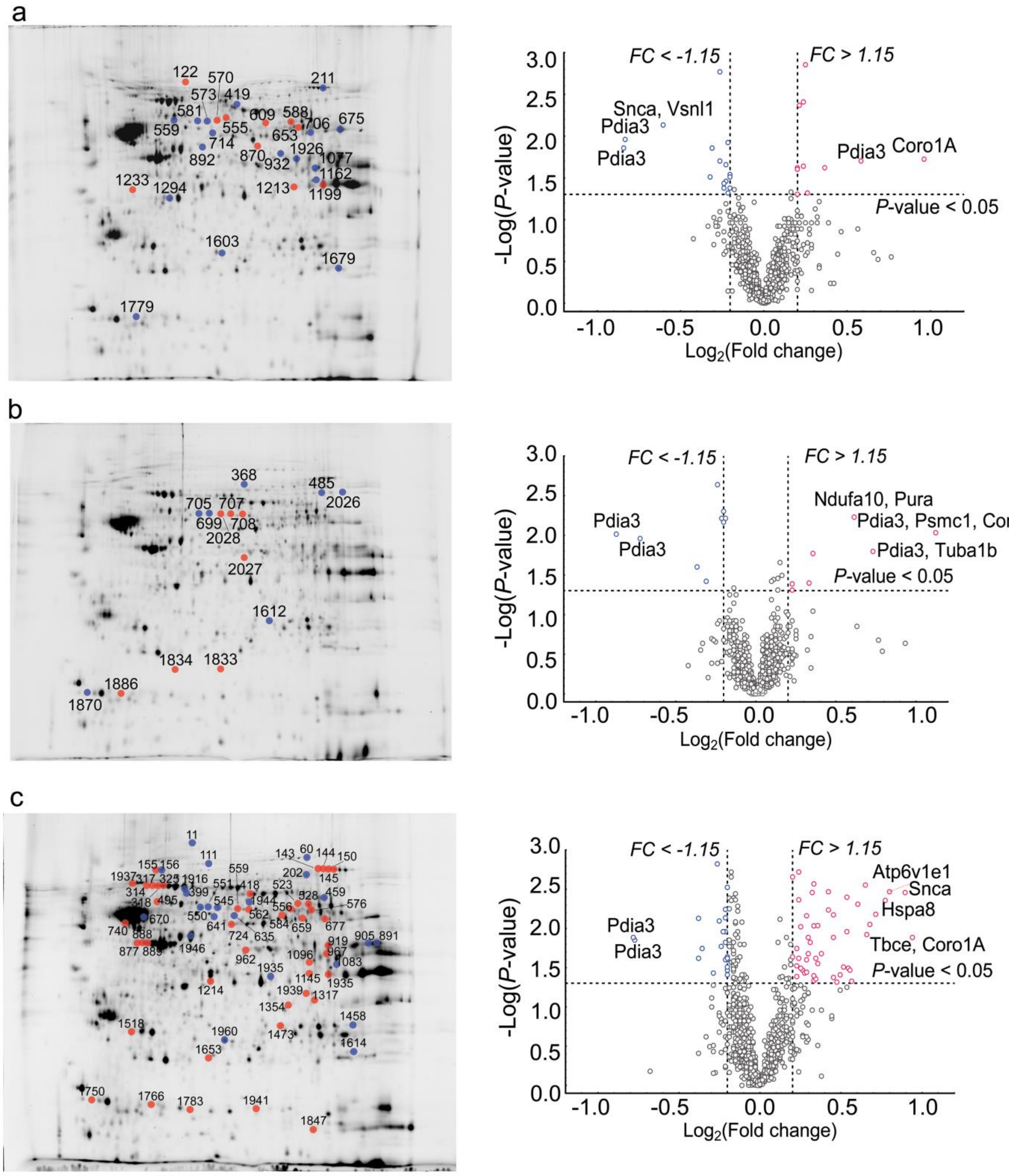

Supplementary Table S1 Significantly altered spots and the proteins identified by LC-MS/MS. Multiple proteins were identified from most spots. Here, only the major components are listed, the distinction is based on the significantly higher unique peptide and PSM numbers. Both the listed Mol. mass and pI values are based on the database sequence that may be significantly different from the mature/active protein, either because of enzymatic processing or other post-translational modifications or it also may be a splice variant. Identical assignments for multiple spots may indicate either one of these options. (PSM: Peptide to spectrum matches)

.raw file uploaded to MassIVE	Spot number	Fold change C/T	*P*-value	Accession number	Protein name	Gene name	Mol. mass (kDa)	pI	No. of unique peptides	No. of PSMs	Seq. cov. (%)
**Frontal cortex**											
180427_18	122	1.18	0.004	D4A133	ATPase H+-transporting V1 subunit A	Atp6v1a	68.2	5.4	10	16	21
				P63018	Heat shock cognate 71 kDa protein	Hspa8	70.8	5.4	10	11	17
180427_13	211	-1.17	0.035	Q9ER34	Aconitate hydratase, mitochondrial	Aco2	85.4	7.9	31	169	41
180427_11	419	-1.15	0.029	P02770	Serum albumin	Alb	68.2	6.1	21	93	33
				P47942	Dihydropyrimidinase-related protein 2	Dpysl2	62.2	6	8	12	17
180427_09	555	1.95	0.019	Q91ZN1	Coronin-1A	Coro1a	51	6.1	11	25	28
180427_14	559	-1.18	0.037	P63039	60 kDa heat shock protein, mitochondrial	Hspd1	60.9	5.9	40	202	60
180427_02	570	1.5	0.02	P11598	Protein disulfide-isomerase A3	Pdia3	56.6	5.9	21	72	46
180427_10	573	-1.78	0.011	P11598	Protein disulfide-isomerase A3	Pdia3	56.6	5.9	30	111	53
180427_17	581	-1.79	0.014	P11598	Protein disulfide-isomerase A3	Pdia3	56.6	5.9	25	62	46
180427_04	588	1.29	0.024	P11980	Pyruvate kinase	Pkm	57.8	6.6	49	254	64
180427_27	609	1.19	0.013	P11980	Pyruvate kinase	Pkm	57.8	6.6	19	37	40
				P61765	Syntaxin-binding protein 1	Stx1bp	67.5	6.5	12	26	21
180427_26	653	1.15	0.024	Q08163	Adenylyl cyclase-associated protein 1	Cap1	51.6	7.2	14	24	29
180427_35	675	-1.2	0.02	P15999	ATP synthase subunit alpha, mitochondrial	Atp5f1a	59.7	9.2	43	249	57
180427_2	706	-1.24	0.014	P10860	Glutamate dehydrogenase 1, mitochondrial	Glud1	61.4	8.1	39	183	58
				P15999	ATP synthase subunit alpha, mitochondrial	Atp5f1a	59.7	9.2	24	45	44

180427_05	714	-1.16	0.049	Q01205	Dihydrolipoyllysine-residue succinyltransferase component of 2-oxoglutarate dehydrogenase complex	Dlst	48.9	8.9	13	52	25
				A0A0G2JTG7	Heterogeneous nuclear ribonucleoprotein H	Hnrnph1	49.2	N.A	13	21	31
180427_12	870	1.16	0.004	Q6AZ26	C-terminal binding protein 1	Ctbp1	42.6	6.1	20	158	52
				F1LMZ8	26S proteasome non-ATPase regulatory subunit 11	Psmd11	47.4	6.1	13	18	36
180427_01	892	1.15	0.05	P47971	Neuronal pentraxin-1	Nptx1	47.2	6.3	10	20	26
				G3V7L6	26S proteasome regulatory subunit 7	Psmc2	48.6	N.A	9	15	25
180427_33	932	-1.16	0.021	P85834	Elongation factor Tu, mitochondrial	Tufm	49.5	7.2	28	105	56
180427_22	1077	-1.16	0.041	P09117	Fructose-bisphosphate aldolase C	Aldoc	39.3	6.7	28	179	69
180427_20	1162	-1.18	0.042	P09117	Fructose-bisphosphate aldolase C	Aldoc	39.3	6.7	23	104	58
180427_34	1199	1.18	0.048	P04797	Glyceraldehyde-3-phosphate dehydrogenase	Gapdh	35.8	8.1	22	99	56
180427_06	1213	1.2	0.048	P04797	Glyceraldehyde-3-phosphate dehydrogenase	Gapdh	35.8	8.1	16	53	34
180427_29	1233	1.15	0.025	Q5XFW8	Protein SEC13 homolog	Sec13	35.5	5.1	4	5	21
				Q9QYU4	Ketimine reductase mu-crystallin	Crym	33.5	5.3	4	5	10
180427_25	1294	-1.25	0.031	P54311	Guanine nucleotide-binding protein G(I)/G(S)/G(T) subunit beta-1	Gnb1	37.3	5.6	14	45	51
180427_03	1603	-1.15	0.042	P62994	Growth factor receptor-bound protein 2	Grb2	25.2	5.9	19	148	76
180427_19	1679	-1.16	0.031	P07895	Superoxide dismutase [Mn], mitochondrial	Sod2	24.7	9	8	63	35
				P39069	Adenylate kinase isoenzyme 1	Ak1	21.6	7.7	8	22	50
				D4A0T0	NADH:ubiquinone oxidoreductase subunit B10	Ndufb10	20.8	7.6	6	13	44
180427_21	1779	-1.52	0.007	P37377	Alpha-synuclein	Snca	14.5	4.7	4	13	34
				P62762	Visinin-like protein 1	Vsnl1	22.1	5	4	5	26
180427_30	1926	-1.2	0.002	P41562	Isocitrate dehydrogenase [NADP] cytoplasmic	Idh1	46.7	6.5	25	73	50
				P09606	Glutamine synthetase	Glul	42.3	6.6	14	63	49
**Hippocampus**											
180628_12	176	-1.16	0.006	P21575	Dynamin-1	Dnm1	97.3	6.4	76	125	67

180628_05	368	-1.15	0.005	P31977	Ezrin	Ezr	69.4	5.8	60	126	69
				Q9QUL6	Vesicle-fusing ATPase	Nsf	82.6	6.5	45	69	69
				P09951	Synapsin-1	Syn1	74	9.8	36	64	57
				O35303	Dynamin-1-like protein	Dnm1l	83.9	6.6	34	41	41
180628_13	485	-1.15	0.007	Q63537	Synapsin-2	Syn2	63.5	8.7	64	192	76
180628_22	699	-1.65	0.011	P11598	Protein disulfide-isomerase A3	Pdia3	56.6	5.9	45	95	67
180628_25	705	-1.83	0.01	P11598	Protein disulfide-isomerase A3	Pdia3	56.6	5.9	41	95	64
180628_18	707	2.18	0.009	P11598	Protein disulfide-isomerase A3	Pdia3	56.6	5.9	49	60	72
				P62193	26S proteasome regulatory subunit 4	Psmc1	49.2	5.9	35	132	59
				Q91ZN1	Coronin-1A	Coro1a	51.1	6.1	25	47	48
180628_03	708	1.66	0.016	P11598	Protein disulfide-isomerase A3	Pdia3	56.6	5.9	45	83	66
				Q6P9V9	Tubulin alpha-1B chain	Tuba1b	50.2	5.9	30	63	64
180628_20	1612	-1.24	0.038	P25113	Phosphoglycerate mutase 1	Pgam1	28.8	6.7	26	67	83
180628_02	1833	1.17	0.041	B5DFF7	Dusp3 protein	Dusp3	20.5	6.1	22	99	88
180628_04	1834	1.28	0.017	D4A1B8	Dynactin subunit 3	Dctn3	21.1	5.6	24	107	88
180628_10	1870	-1.29	0.025	P37377	Alpha-synuclein	Snca	14.5	4.7	16	41	69
180628_06	1886	1.17	0.049	Q5PQN0	Neurocalcin-delta	Ncald	22.2	5.2	22	49	80
180628_14	2026	-1.18	0.002	Q63537	Synapsin-2	Syn2	63.5	8.7	51	131	70
180628_01	2027	1.53	0.006	Q561S0	NADH dehydrogenase [ubiquinone] 1 alpha subcomplex subunit 10, mitochondrial	Ndufa10	40.5	7.6	24	52	64
				F1LPS8	Transcriptional activator protein Pur-alpha	Pura	31.9	8.7	25	44	65
180628_19	2028	1.26	0.04	P11598	Protein disulfide-isomerase A3	Pdia3	56.6	5.9	24	38	45
**Occipital cortex**											
180508_22	11	-1.22	0.037	A0A0G2JZ69	Spectrin alpha chain, non-erythrocytic 1	Sptan1	286.7	5.3	26	33	15
180522_20	60	-1.16	0.015	P21575	Dynamin-1	Dnm1	97.3	6.4	86	168	70
180508_11	111	-1.15	0.025	Q68FP1	Gelsolin	Gsn	86.1	5.8	24	74	38
180525_03	143	1.18	0.024	Q9ER34	Aconitate hydratase, mitochondrial	Aco2	85.4	7.9	56	122	65
180522_16	144	1.34	0.006	Q9ER34	Aconitate hydratase, mitochondrial	Aco2	85.4	7.9	58	113	67
				Q99PF5	Far upstream element-binding protein 2	Khrsp	74	6.4	41	64	54

				P09951	Synapsin-1	Syn1	74	9.8	34	55	57
180516_28	145	1.26	0.009	Q9ER34	Aconitate hydratase, mitochondrial	Aco2	85.4	7.9	63	148	68
180508_05	150	1.22	0.025	Q9ER34	Aconitate hydratase, mitochondrial	Aco2	85.4	7.9	58	210	66
180508_12	155	1.18	0.004	A0A0G2JVH4	MICOS complex subunit MIC60	Immt	86.2	5.6	23	34	34
180522_08	156	-1.2	0.002	P46462	Transitional endoplasmic reticulum ATPase	Vcp	89.3	5.1	59	93	60
				A0A0G2JVH4	MICOS complex subunit MIC60	Immt	86.2	5.6	41	66	48
180508_25	202	-1.16	0.011	P09951	Synapsin-1	Syn1	74	9.8	27	51	39
				Q5BJU7	Wiskott-Aldrich syndrome protein family member 1	Wasf1	61.5	5.9	14	31	32
				Q9QUL6	Vesicle-fusing ATPase	Nsf	82.7	6.5	17	27	30
180516_19	317	1.64	0.007	P63018	Heat shock cognate 71 kDa protein	Hspa8	70.9	5.4	48	103	61
180522_12	318	1.71	0.004	P63018	Heat shock cognate 71 kDa protein	Hspa8	70.9	5.4	50	120	59
180508_09	325	1.52	0.005	P63018	Heat shock cognate 71 kDa protein	Hspa8	70.9	5.4	48	152	62
				D4A133	ATPase H+-transporting V1 subunit A	Atp6v1a	68.3	5.4	18	23	31
				P23565	Alpha-internexin	Ina	56.1	5.2	17	21	43
180516_04	399	-1.15	0.023	P23565	Alpha-internexin	Ina	56.1	5.2	18	23	42
180516_29	418	1.34	0.003	P47942	Dihydropyrimidinase-related protein 2	Dpysl2	62.3	6	46	106	76
180516_18	459	-1.18	0.021	Q9JHU0	Dihydropyrimidinase-related protein 5	Dpysl5	61.5	6.6	48	87	75
				Q62951	Dihydropyrimidinase-related protein 4 (Fragment)	Dpysl4	61.1	6.3	41	73	80
				Q62950	Dihydropyrimidinase-related protein 1	Crmp1	62.2	6.6	30	45	66
180522_21	495	1.57	0.003	P23565	Alpha-internexin	Ina	56.1	5.2	44	115	74
				Q6P9V9	Tubulin alpha-1B chain	Tuba1b	50.2	4.9	20	38	49
				P61980	Heterogeneous nuclear ribonucleoprotein K	Hnrnpk	51	5.4	16	21	40
180508_18	523	1.37	0.013	Q68FS4	Cytosol aminopeptidase	Lap3	56	6.8	42	91	72
				P85845	Fascin	Fscn1	54.5	6.3	36	81	73
				Q6P6R2	Dihydrolipoyl dehydrogenase, mitochondrial	Dld	54	8	24	64	62
				Q68FZ8	Propionyl coenzyme A carboxylase, beta polypeptide	Pccb	58.7	8	22	31	51
				Q63537	Synapsin-2	Syn2	63.5	8.7	19	37	37

180516_09	528	1.45	0.011	P11980	Pyruvate kinase PKM	Pkm	57.8	6.6	72	193	87
180516_17	545	-1.71	0.014	P11598	Protein disulfide-isomerase A3	Pdia3	56.6	5.9	40	92	64
180516_20	550	-1.3	0.024	P47942	Dihydropyrimidinase-related protein 2	Dpysl2	62.3	6	56	141	83
180522_03	551	-1.72	0.013	P11598	Protein disulfide-isomerase A3	Pdia3	56.6	5.9	33	63	58
				P62815	V-type proton ATPase subunit B, brain isoform	Atp6v1b2	56.6	5.6	18	25	39
180508_19	556	1.26	0.003	Q63537	Synapsin-2	Syn2	63.5	8.7	28	73	44
				Q6P6R2	Dihydrolipoyl dehydrogenase, mitochondrial	Dld	54	8	19	32	53
				Q68FZ8	Propionyl coenzyme A carboxylase, beta polypeptide	Pccb	58.7	8	21	34	53
				D3ZQ02	WD Repeat Domain 37	Wdr37	49.9	6.8	15	31	31
				Q68FS4	Cytosol aminopeptidase	Lap3	56.2	6.8	18	29	44
				P85845	Fascin	Fscn1	54.5	6.3	13	17	40
				P21575	Dynamin-1	Dnm1	97.3	6.4	18	21	24
180516_26	559	1.92	0.013	Q5FVQ9	Tubulin-specific chaperone E	Tbce	59	5.9	33	47	72
				Q91ZN1	Coronin-1A	Coro1a	51.1	6.1	37	31	58
180516_10	562	1.43	0.031	Q91ZN1	Coronin-1A	Coro1a	51.1	6.1	37	100	58
180522_07	584	1.15	0.002	O08651	D-3-phosphoglycerate dehydrogenase	Phgdh	56.5	6.3	45	87	66
				Q63537	Synapsin-2	Syn2	63.5	8.7	36	66	59
				D3ZQ02	WD repeat-domain 37	Wdr37	49.8	6.8	22	44	53
180508_30	635	-1.16	0.025	A0A0G2K7G7	Septin-8	Sep8	51.3	5.7	8	15	25
				P85845	Fascin	Fscn1	54.5	6.3	5	6	17
				Q5XIM9	T-complex protein 1 subunit beta	Cct2	57.5	6	4	5	10
				P29066	Beta-arrestin-1	Arrb1	47	5.9	4	5	13
180516_27	641	-1.16	0.007	B0BNF1	Septin-8	Sep8	55.8	5.8	14	17	42
				Q4QQV4	Dead end homolog 1 (Zebrafish)	Hars	57.4	5.9	13	14	33
180516_03	659	1.28	0.028	P11980	Pyruvate kinase PKM	Pkm	57.8	6.6	54	87	78
				B2GV06	Succinyl-CoA:3-ketoacid coenzyme A transferase 1, mitochondrial	Oxct1	56.2	8.7	23	37	41
				Q08163	Adenylyl cyclase-associated protein 1	Cap1	51.6	7.2	23	35	53
180508_17	670	-1.15	0.024	P85108	Tubulin beta-2A chain	Tubb2a	49.9	4.8	62	380	76

				Q6P9V9	Tubulin alpha-1B chain	Tuba1b	50.2	4.9	32	113	68
				Q9Z0W5	Protein kinase C and casein kinase substrate in neurons protein 1	Pacsin1	50.4	5.2	31	62	64
180516_12	677	1.23	0.021	P15999	ATP synthase subunit alpha, mitochondrial	Atp5a1	59.8	9.2	53	112	74
				Q4V8I9	UDP-glucose pyrophosphorylase 2	Ugp2	57	7.2	49	99	64
180516_14	707	-1.15	0.004	Q9Z0W5	Protein kinase C and casein kinase substrate in neurons protein 1	Pacsin1	50.4	5.2	51	95	76
				Q6AYH5	Dynactin subunit 2	Dctn2	44.1	5.1	27	34	60
180522_05	724	1.39	0.048	P04764	Alpha-enolase	Eno1	47.1	6.2	45	126	84
180522_29	740	1.26	0.04	P07323	Gamma-enolase	Eno2	47.1	5	59	300	94
180508_26	809	1.17	0.041	P20650	Protein phosphatase 1A	Ppm1a	42.4	5.2	24	51	59
				Q9Z214	Homer protein homolog 1	Homer1	41.3	5.4	16	26	44
				P07335	Creatine kinase B-type	Ckb	42.7	5.4	13	24	39
				P63259	Actin, cytoplasmic 2	Actg1	41.8	5.3	15	32	46
				Q5VLR5	BWK4	Erp44	46.9	5.1	9	32	32
				Q3S4A4	ADP-ribosylation factor GTPase activating protein 1 heart isoform	Arfgap1	46.7	5.4	16	23	41
				P19527	Neurofilament light polypeptide	Nefl	61.3	4.6	12	14	25
				P10719	ATP synthase subunit beta, mitochondrial	Atp5b	56.4	5.2	10	13	25
180516_21	877	1.28	0.009	P60711	Actin, cytoplasmic 1	Actb	41.7	5.3	52	105	86
				A0A0G2JSU4	N-myc downstream regulated gene 2, isoform CRA_b	Ndrg2	39.3	5.3	38	69	82
180508_06	888	1.6	0.009	P60711	Actin, cytoplasmic 1	Actb	41.7	5.3	49	121	73
180516_22	889	1.37	0.042	P63259	Actin, cytoplasmic 2	Actg1	41.8	5.3	46	130	84
				A0A0G2JSU4	N-myc downstream regulated gene 2, isoform CRA_b	Ndrg2	39.3	5.3	27	44	70
180508_04	891	-1.18	0.001	P16617	Phosphoglycerate kinase 1	Pgk1	44.5	8	46	158	82
				P05065	Fructose-bisphosphate aldolase A	Aldoa	39.4	8.3	18	33	50
				G3V936	Citrate synthase	Cs	51.8	8.6	18	23	41
180508_03	905	-1.19	0.008	P16617	Phosphoglycerate kinase 1	Pgk1	44.5	8	41	118	85
				P05065	Fructose-bisphosphate aldolase A	Aldoa	39.4	8.3	29	68	77
				Q5BJT9	Creatine kinase U-type, mitochondrial	Ckmt1	47	8.6	20	61	47

				P04797	Glyceraldehyde-3-phosphate dehydrogenase	Gapdh	35.8	8.1	14	26	46
				G3V936	Citrate synthase	Cs	51.8	8.6	17	25	31
				Q68FX0	Isocitrate dehydrogenase [NAD] subunit beta, mitochondrial	Idh3B	42.4	8.9	14	22	31
180525_12	919	1.23	0.013	P16617	Phosphoglycerate kinase 1	Pgk1	44.5	8	37	71	79
				Q4FZZ4	Pyruvate dehydrogenase E1 component subunit alpha	Pdha1	43.2	8.5	30	54	67
180508_21	962	1.3	0.021	D3ZDH8	Septin-5	Sept5	43.9	6.2	30	102	63
				Q91Y81	Septin-2	Sept2	41.6	6.1	9	16	36
180522_27	967	1.2	0.034	P16617	Phosphoglycerate kinase 1	Pgk1	44.5	8	40	87	81
				P26284	Pyruvate dehydrogenase E1 component subunit alpha, somatic form, mitochondrial	Pdha1	43.2	8.5	28	48	60
180522_24	1083	-1.15	0.034	P09117	Fructose-bisphosphate aldolase C	Aldoc	39.3	6.7	49	164	96
180508_10	1096	1.47	0.034	P09117	Fructose-bisphosphate aldolase C	Aldoc	39.3	6.7	24	43	62
				F1LMH0	Neuronal-specific septin-3	Sept3	40.7	6.8	21	47	55
180508_27	1145	1.41	0.039	P09117	Fructose-bisphosphate aldolase C	Aldoc	39.3	6.7	24	41	60
180525_05	1214	1.58	0.012	O88989	Malate dehydrogenase, cytoplasmic	Mdh1	36.5	6.2	20	58	71
180522_15	1317	1.21	0.037	Q9Z2L0	Voltage-dependent anion-selective channel protein 1	Vdac1	30.8	8.6	29	90	91
				Q497B0	Omega-amidase NIT2	Nit2	30.7	6.9	11	16	50
180508_06	1354	1.74	0.003	G3V7L8	ATPase, H+ transporting, V1 subunit E isoform 1, isoform CRA_a	Atp6v1e1	26.1	8.4	23	56	65
180508_14	1458	-1.16	0.015	Q6AYK6	Calcyclin-binding protein	Cacybp	26.5	7.6	19	56	78
				P11348	Dihydropteridine reductase	Qdpr	25.6	7.7	15	24	56
				P27139	Carbonic anhydrase 2	Ca2	29.1	6.9	13	31	55
				P08009	Glutathione S-transferase Yb-3	Gstp1	25.7	6.9	11	16	55
180522_13	1473	1.25	0.004	P25113	Phosphoglycerate mutase 1	Pgam1	28.8	6.7	26	82	82
				D3ZUX5	MICOS complex subunit	Chchd3	26.4	8.2	15	23	44
180522_11	1518	1.19	0.01	P62747	Rho-related GTP-binding protein RhoB	Rhob	22.1	5.1	11	25	55
				Q00981	Ubiquitin carboxyl-terminal hydrolase isozyme L1	Uchl1	24.8	5.1	12	16	49
				Q5XI73	Rho GDP-dissociation inhibitor 1	Arhgdia	23.4	5.1	9	13	40

180508_28	1614	-1.17	0.011	P04906	Glutathione S-transferase P	Gstp1	23.4	6.9	18	69	68
180508_13	1653	1.19	0.032	P39069	Adenylate kinase isoenzyme 1	Ak1	21.6	7.7	7	10	50
				P31399	ATP synthase subunit d, mitochondrial	Atp5h	18.8	6.2	6	12	42
180522_19	1750	1.86	0.004	P37377	Alpha-synuclein	Snca	14.5	4.7	15	28	69
				Q9QXU9	ProSAAS	Pcsk1n	27.4	5.7	9	17	39
180508_29	1766	1.26	0.04	P04906	Glutathione S-transferase P	Gstp1	23.4	6.9	6	11	42
				P13668	Stathmin	Stmn1	17.3	5.8	4	6	30
180525_07	1783	1.17	0.018	P41498	Low molecular weight phosphotyrosine protein phosphatase	Acp1	18.2	6.1	9	15	70
				P13668	Stathmin	Stmn1	17.3	5.8	6	12	34
180508_02	1847	1.37	0.007	P10111	Peptidyl-prolyl cis-trans isomerase A	Ppia	17.9	8.3	4	8	43
180516_30	1916	-1.15	0.003	P63018	Heat shock cognate 71 kDa protein	Hspa8	70.9	5.4	62	229	72
				D4A133	ATPase H+-transporting V1 subunit A	Atp6v1a	68.3	5.4	49	118	69
				P23565	Alpha-internexin	Ina	56.1	5.2	38	111	70
180525_04	1935	-1.28	0.018	P63088	Serine/threonine-protein phosphatase PP1-gamma catalytic subunit	Ppp1cc	37	6.1	18	36	60
180522_23	1936	1.27	0.024	P04797	Glyceraldehyde-3-phosphate dehydrogenase	Gapdh	35.8	8.1	35	100	75
180522_06	1937	1.18	0.002	P06761	78 kDa glucose-regulated protein	Hspa5	72.3	5.1	42	82	51
				D4AA63	Ubiquilin 2	Ubqln2	67.3	5.2	25	41	27
180522_04	1939	1.45	0.004	P04797	Glyceraldehyde-3-phosphate dehydrogenase	Gapdh	35.8	8.1	30	49	58
				B5DEZ6	Glucosamine-6-phosphate isomerase	Gnpda2	31	6.3	15	23	62
180522_14	1941	1.52	0.005	P45592	Cofilin-1	Cfl1	18.5	8.2	13	31	78
				B0BN18	Prefoldin subunit 2	Pfdn2	16.6	6.2	9	15	56
180516_02	1944	-1.16	0.006	P47942	Dihydropyrimidinase-related protein 2	Dpysl2	62.3	6	38	68	70
				D3ZAA9	MAGUK p55 subfamily member 2	Mpp2	61.6	6	35	58	60
180522_30	1946	-1.15	0.031	P07335	Creatine kinase B-type	Ckb	42.7	5.4	46	380	79
				Q9JKB7	Guanine deaminase	Gda	50.9	5.5	47	130	83
180525_14	1960	-1.3	0.007	O35244	Peroxiredoxin-6	Prdx6	24.8	5.6	25	106	79

Supplementary Table S2 List of functionally clustered significantly altered proteins and their cellular localization

Accesion number	Protein name	Gene name	Subcellular location
**Frontal cortex**			
**Energy and carbohydrate metabolism**			
Q9ER34	Aconitate hydratase, mitochondrial	Aco2	Mitochondria
P09117	Fructose-bisphosphate aldolase C	Aldoc	Cytoplasm
P04797	Glyceraldehyde-3-phosphate dehydrogenase	Gapdh	Cytoplasm
P11980	Pyruvate kinase PKM	Pkm	Cytoplasm
Q01205	Dihydrolipoyllysine-residue succinyltransferase component of 2-oxoglutarate dehydrogenase complex	Dlst	Mitochondria
P10860	Glutamate dehydrogenase 1, mitochondrial	Glud1	Mitochondria
P41562	Isocitrate dehydrogenase [NADP] cytoplasmic	Idh1	Cytoplasm
P15999	ATP synthase subunit alpha, mitochondrial	Atp5f1a	Mitochondria
D4A0T0	NADH:ubiquinone oxidoreductase subunit B10	Ndufb10	Mitochondria
**Protein turnover**			
F1LMZ8	26S proteasome non-ATPase regulatory subunit 11	Psmd11	Cytoplasm/Nucleus
G3V7L6	26S proteasome regulatory subunit 7	Psmc2	Cytoplasm
P63039	60 kDa heat shock protein, mitochondrial	Hspd1	Mitochondria
P63018	Heat shock cognate 71 kDa protein	Hspa8	Nucleus/Plasma membrane
P11598	Protein disulfide-isomerase A3	Pdia3	Endoplasmic reticulum/ Nucleus/ Extracellular region
Q5XFW8	Protein SEC13 homolog	Sec13	Cytoplasm/ ER/ Golgi apparatus/ Lysosome/ Nucleus
**Signal transduction**			
P54311	Guanine nucleotide-binding protein G(I)/G(S)/G(T) subunit beta-1	Gnb1	Plasma membrane
P62994	Growth factor receptor-bound protein 2	Grb2	Plasma membrane/ Cytoplasm/ Nucleus/ Endosome
P62762	Visinin-like protein 1	Vsnl1	Cytoplasm
Q91ZN1	Coronin-1A	Coro1a	Cytoskeleton/ Cytoplasm/ Endosome
**Synaptic transmision**			
P37377	Alpha-synuclein	Snca	Nucleus/ Cytosol/ Extracellular region
P61765	Syntaxin-binding protein 1	Stx1bp	Plasma membrane/ Cytoplasm
P47971	Neuronal pentraxin-1	Nptx1	Mitochondria/ Plasma membrane
**Respons to oxidative stress**			

Q6AZ26	C-terminal binding protein 1	Ctbp1	Nucleus
P07895	Superoxide dismutase [Mn], mitochondrial	Sod2	Mitochondria
**Amino acid and nucleotide metabolism**			
P09606	Glutamine synthetase	Glul	Mitochondria
P39069	Adenylate kinase isoenzyme 1	Ak1	Cytoplasm
**Cytoskelatal proteins**			
P47942	Dihydropyrimidinase-related protein 2	Dpysl2	Cytoplasm/ Membrane
Q08163	Adenylyl cyclase-associated protein 1	Cap1	Cytoskeleton/Plasma membrane
**Ion transport**			
D4A133	ATPase H+-transporting V1 subunit A	Atp6v1a	Plasma membrane/ Lysosome/ Cytoplasm
**RNA processing**			
Q8VHV7	Heterogeneous nuclear ribonucleoprotein H	Hnrnph1	Nucleus
**Mitochondrial dynamics**			
P85834	Elongation factor Tu, mitochondrial	Tufm	Mitochondria
Q9QYU4	Ketimine reductase mu-crystallin	Crym	Cytoplasm
**Other**			
P02770	Serum albumin	Alb	Extracellular region
**Hippocampus**			
**Energy and carbohydrate metabolism**			
P25113	Phosphoglycerate mutase 1	Pgam1	Cytoplasm
Q561S0	NADH dehydrogenase [ubiquinone] 1 alpha subcomplex subunit 10, mitochondrial	Ndufa10	Mitochondria
**Cytoskelatal proteins**			
Q6P9V9	Tubulin alpha-1B chain	Tuba1b	Cytoskeleton
P31977	Ezrin	Ezr	Cytoskeleton/ Plasma membrane
**Protein turnover**			
P62193	26S proteasome regulatory subunit 4	Psmc1	Cytoskeleton/ Nucleus
P11598	Protein disulfide-isomerase A3	Pdia3	Endoplasmic reticulum/ Nucleus/ Extracellular region
**Signal transduction**			
B5DFF7	Dusp3 protein	Dusp3	Cytoplasm
Q5PQN0	Neurocalcin-delta	Ncald	Cytoplasm
Q91ZN1	Coronin-1A	Coro1a	Cytoskeleton/ Cytoplasm
**Synaptic transmision**			
Q9QUL6	Vesicle-fusing ATPase	Nsf	Golgi apparatus
P37377	Alpha-synuclein	Snca	Nucleus/ Cytoplasm/ Extracellular region
P09951	Synapsin-1	Syn1	Plasma membrane
Q63537	Synapsin-2	Syn2	Plasma membrane
**Vesicle transport and endocytosis**			
D4A1B8	Dynactin subunit 3	Dctn3	Cytoplasm/Cytoskeleton

O35303	Dynamin-1-like protein	Dnm1l	Cytoplasm/Mitochondria
P21575	Dynamin-1	Dnm1	Cytoskeleton
**Transcription and RNA processing**			
F1LPS8	Transcriptional activator protein Pur-alpha	Pura	Nucleus
**Occipital cortex**			
**Carbohydrate and energy metabolism**			
B5DEZ6	Glucosamine-6-phosphate isomerase	Gnpda2	Cytoplasm
P26284	Pyruvate dehydrogenase E1 component subunit alpha, somatic form, mitochondrial	Pdha1	Mitochondrial matrix
Q4V8I9	UDP-glucose pyrophosphorylase 2	Ugp2	Cytoplasm
P04764	Alpha-enolase	Eno1	Plasma membrane/Cytoplasm
P05065	Fructose-bisphosphate aldolase A	Aldoa	Cytoplasm
P09117	Fructose-bisphosphate aldolase C	Aldoc	Cytoplasm
P07323	Gamma-enolase	Eno2	Plasma membrane/Cytoplasm
P04797	Glyceraldehyde-3-phosphate dehydrogenase	Gapdh	Cytoplasm
P16617	Phosphoglycerate kinase 1	Pgk1	Cytoplasm
P25113	Phosphoglycerate mutase 1	Pgam1	Cytoplasm/Nucleus
P11980	Pyruvate kinase PKM	Pkm	Cytoplasm
Q9ER34	Aconitate hydratase, mitochondrial	Aco2	Mitochondria
G3V936	Citrate synthase	Cs	Mitochondria
Q6P6R2	Dihydrolipoyl dehydrogenase, mitochondrial	Dld	Mitochondria
Q68FX0	Isocitrate dehydrogenase [NAD] subunit beta, mitochondrial	Idh3B	Mitochondria
O88989	Malate dehydrogenase, cytoplasmic	Mdh1	Cytoplasm
P39069	Adenylate kinase isoenzyme 1	Ak1	Cytoplasm
P15999	ATP synthase subunit alpha, mitochondrial	Atp5a1	Mitochondria
P10719	ATP synthase subunit beta, mitochondrial	Atp5b	Mitochondria
P31399	ATP synthase subunit d, mitochondrial	Atp5h	Mitochondria
P07335	Creatine kinase B-type	Ckb	Cytoplasm
Q5BJT9	Creatine kinase U-type, mitochondrial	Ckmt1	Mitochondria
**Cytoskelatal proteins and neurite outgrowth**			
P60711	Actin, cytoplasmic 1	Actb	Cytoskeleton
P63259	Actin, cytoplasmic 2	Actg1	Cytoskeleton
P85845	Fascin	Fscn1	Cytoskeleton
Q68FP1	Gelsolin	Gsn	Cytoskeleton/Cytoplasm
P19527	Neurofilament light polypeptide	Nefl	Cytoskeleton
Q91Y81	Septin-2	Sept2	Cytoskeleton
B0BNF1	Septin-8	Sept8	Cytoskeleton
P13668	Stathmin	Stmn1	Cytoskeleton
Q6P9V9	Tubulin alpha-1B chain	Tuba1b	Cytoskeleton
P85108	Tubulin beta-2A chain	Tubb2a	Cytoskeleton
P23565	Alpha-internexin	Ina	Cytoskeleton

P45592	Cofilin-1	Cfl1	Cytoskeleton /Plasma membrane/ Nucleus
F1LMH0	Neuronal-specific septin-3	Sept3	Cytoplasm/ Cytoskeleton/ Plasma membrane
B0BN18	Prefoldin subunit 2	Pfdn2	Cytoplasm/ Nucleus
Q08163	Adenylyl cyclase-associated protein 1	Cap1	Cytoskeleton
Q62950	Dihydropyrimidinase-related protein 1	Crmp1	Cytoskeleton/ Cytoplasm
Q9Z0W5	Protein kinase C and casein kinase substrate in neurons protein 1	Pacsin1	Plasma membrane/ Cytoplasm
A0A0G2JZ69	Spectrin alpha chain, non-erythrocytic 1	Sptan1	Cytoskeleton
Q5FVQ9	Tubulin-specific chaperone E	Tbce	Cytoskeleton
Q5BJU7	Wiskott-Aldrich syndrome protein family member 1	Wasf1	Cytoskeleton
P47942	Dihydropyrimidinase-related protein 2	Dpysl2	Plasma membrane/ Cytoplasm/ Cytoskeleton
Q62951	Dihydropyrimidinase-related protein 4 (Fragment)	Dpysl4	Cytoskeleton/ Cytoplasm
Q9JHU0	Dihydropyrimidinase-related protein 5	Dpysl5	Cytoplasm
A0A0G2JSU4	N-myc downstream regulated gene 2, isoform CRA_b	Ndrg2	Cytoplasm/ Plasma membrane
**Protein turnover**			
P06761	78 kDa glucose-regulated protein	Hspa5	Endoplasmic reticulum lumen
Q5VLR5	BWK4	Erp44	Endoplasmic reticulum
Q6AYK6	Calcyclin-binding protein	Cacybp	Nucleus
P63018	Heat shock cognate 71 kDa protein	Hspa8	Nucleus/ Plasma membrane
P10111	Peptidyl-prolyl cis-trans isomerase A	Ppia	Cytosol/ Extracellular region
P11598	Protein disulfide-isomerase A3	Pdia3	Endoplasmic reticulum/ Nucleus/ Extracellular region
Q5XIM9	T-complex protein 1 subunit beta	Cct2	Cytoplasm
D4AA63	Ubiquilin 2	Ubqln2	Cytoplasm
Q00981	Ubiquitin carboxyl-terminal hydrolase isozyme L1	Uchl1	Endoplasmic reticulum
**Synaptic transmision**			
Q9Z214	Homer protein homolog 1	Homer1	Postsynaptic density
D3ZAA9	MAGUK p55 subfamily member 2	Mpp2	Postsynaptic density/ Cytoskeleton/ Dendritic spine membrane
P37377	Alpha-synuclein	Snca	Nucleus/Cytosol/Extracellular region
P41498	Low molecular weight phosphotyrosine protein phosphatase	Acp1	Cytoplasm
D3ZDH8	Septin-5	Sept5	Plasma membrane/ Cell cortex
P09951	Synapsin-1	Syn1	Cytoplasm/ Cytoskeleton
Q63537	Synapsin-2	Syn2	Plasma membrane
Q9QUL6	Vesicle-fusing ATPase	Nsf	Golgi apparatus
**Signal transduction**			

Q3S4A4	ADP-ribosylation factor GTPase activating protein 1 heart isoform	Arfgap1	Postsynaptic density
P63088	Serine/threonine-protein phosphatase PP1-gamma catalytic subunit	Ppp1cc	Nucleus/ Mitochondria
P29066	Beta-arrestin-1	Arrb1	Plasma membrane/ Nucleus
P20650	Protein phosphatase 1A	Ppm1a	Nucleus/ Cytoplasm
Q5XI73	Rho GDP-dissociation inhibitor 1	Arhgdia	Cytoplasm
P62747	Rho-related GTP-binding protein RhoB	Rhob	Cell membrane/ Endosome/ Nucleus
Q91ZN1	Coronin-1A	Coro1a	Cytoskeleton/ Cytoplasm/ Endosome
**Amino acid and nucleotide metabolism**			
Q68FS4	Cytosol aminopeptidase	Lap3	Cytoplasm
O08651	D-3-phosphoglycerate dehydrogenase	Phgdh	Plasma membrane
P11348	Dihydropteridine reductase	Qdpr	Cytoplasm
Q497B0	Omega-amidase NIT2	Nit2	Cytoplasm
Q9JKB7	Guanine deaminase	Gda	Cytoplasm
**Ion transport**			
D4A133	ATPase H+-transporting V1 subunit A	Atp6v1a	Plasma membrane/ Lysosome
G3V7L8	ATPase, H+ transporting, V1 subunit E isoform 1, isoform CRA_a	Atp6v1e1	Plasma membrane/ Endosome/ Cytoplasm
Q9Z2L0	Voltage-dependent anion-selective channel protein 1	Vdac1	Mitochondria/ Plasma membrane
P62815	V-type proton ATPase subunit B, brain isoform	Atp6v1b2	Mitochondria
**Respons to oxidative stress**			
P08009	Glutathione S-transferase Yb-3	Gstm3	Nucleus/ Mitochondria
O35244	Peroxiredoxin-6	Prdx6	Cytoplasm
P04906	Glutathione S-transferase P	Gstp1	Nucleus/ Mitochondria
**Mitochondrial dynamics**			
A0A0G2JVH4	MICOS complex subunit MIC60	Immt	Mitochondria
D3ZUX5	MICOS complex subunit	Chchd3	Mitochondria
Q4QQV4	Dead end homolog 1 (Zebrafish)	Hars	Mitochondria
**Ketone and lipid mechanism**			
B2GV06	Succinyl-CoA:3-ketoacid coenzyme A transferase 1, mitochondrial	Oxct1	Mitochondria
Q68FZ8	Propionyl coenzyme A carboxylase, beta polypeptide	Pccb	Mitochondria
**Vesicle transport and endocytosis**			
P46462	Transitional endoplasmic reticulum ATPase	Vcp	Cytoplasm/ Endoplasmic reticulum
Q6AYH5	Dynactin subunit 2	Dctn2	Cytoskeleton
P21575	Dynamin-1	Dnm1	Cytoskeleton
**Transcription and RNA processing**			
Q99PF5	Far upstream element-binding protein 2	Khrsp	Nucleus/ Cytoplasm

P61980	Heterogeneous nuclear ribonucleoprotein K	Hnrnpk	Nucleus/ Cytoplasm
**Other**			
P27139	Carbonic anhydrase 2	Ca2	Plasma membrane
Q9QXU9	ProSAAS	Pcsk1n	Extracellular region/ Golgi apparatus
